# Alcohol or No Alcohol in Wine: Half a Century of Debate

**DOI:** 10.3390/foods14111854

**Published:** 2025-05-23

**Authors:** Mariantonietta Succi, Francesca Coppola, Bruno Testa, Michela Pellegrini, Massimo Iorizzo

**Affiliations:** 1 Department of Agricultural, Environmental and Food Sciences, University of Molise, Via De Sanctis, 86100 Campobasso, Italy; bruno.testa@unimol.it (B.T.); iorizzo@unimol.it (M.I.); 2 Department of Agricultural Sciences, University of Naples “Federico II”, Piazza Carlo di Borbone 1, 80055 Portici, Italy; francesca.coppola2@unina.it; 3 Department of Agricultural, Food, Environmental and Animal Science, University of Udine, Via Sondrio 2A, 33100 Udine, Italy

**Keywords:** alcohol abuse, red wine, polyphenols, wine market, low-alcohol wine, dealcoholised wine

## Abstract

Alcoholic beverages have been consumed for centuries in different countries around the world. Today, we know that the ethanol they contain is associated with significant health risks, especially in the case of abuse, in individuals with special health conditions, and in pregnant women. However, over the years, awareness has grown that wine, especially red wine, has a beneficial effect on human health due to the powerful effect of the antioxidant substances it contains, known under the generic term of polyphenols. The main concern remains around the ethanol content of wine and its effects on health. After fifty years of research and studies, the debate is still open, with conflicting indications about the positive effect of moderate wine consumption in the context of a balanced diet and the toxic effect of ethanol even in low doses. In this disputed area, the market for low-alcohol and alcohol-free wines has found its place in the last decade, creating a new opportunity for the global wine trade. These new types of wine are going to open a new chapter in the history of wine. In this review, we have summarised the main aspects of the health implications of wine consumption considering scientific evidence from the last 50 years, including low-alcohol and dealcoholised wine.

## 1. Introduction

Nowadays, wine is one of the main traditional alcoholic beverages consumed all over the world due to its easy accessibility, preservability, legality, and diffusion. It is mainly used for hedonic reasons. Its beneficial effects have been recognised since antiquity, and its use in medical therapy is well-documented. Wine, for example, was mixed with honey for coughs, and various unguents containing wine and herbs were used for skin diseases [1]; it was used for asthma, constipation, epilepsy, and jaundice [2]. Wine was also employed as an antiseptic, sedative, and anaesthetic due to its alcohol content [3].

The role of wine in maintaining a good health status is still a hot topic today. It contains numerous bioactive molecules, including polyphenols, flavonoids, flavonols, anthocyanins, proanthocyanidins, and stilbenes, recognised for their beneficial health properties, such as reducing the risk of cardiovascular infractions, neurological diseases, atherosclerosis, and all causes of mortality [4]. Unfortunately, despite massive information campaigns on the risks of alcohol abuse, its over-consumption is estimated at 76.3 million people worldwide, with almost 1.8 million deaths each year [5]. Moreover, alcohol is casually linked to several cancers and considered a carcinogen, with a high variability in individual responses influenced by factors like genetics, diet, and overall health [6].

In this context, dealcoholised wine has the potential to attract different market segments and meet the needs of those consumers who are more health-conscious or simply cannot drink alcoholic beverages due to medical advice [7]. The question is whether dealcoholised wine really is the panacea for all ills or whether, rather, it may pose health issues to the consumer. The following paragraphs illustrate the pros and cons of wine consumption described from 1970 to the present. The last paragraphs are devoted to low-alcohol and dealcoholised wine and include a description of the target market, the characteristics of these wines in relation to their stability, and some considerations regarding health properties.

## 2. Search Methodology

The Scopus database was queried for basic and clinical articles published from 1970 to the present. After several attempts, the terms “Wine AND Health” were chosen as the best in terms of the breadth of topics covered. English language restriction was imposed. The search yielded 6812 references, including original articles and reviews.

Considering the enormous amount of data obtained, the search was repeated in two different periods: the first from the year 1970 to 1999 and the second from the year 2000 to 2024. Co-occurrence analysis of terms in titles, abstracts, and keywords of documents obtained in Scopus from 1970 to 1999 and from 2000 to 2024 was performed using VOSviewer (VOSviewer version 1.6.20). This strategy allowed us to identify the main themes addressed by researchers in the two periods under consideration. Full-text articles were revised to determine their interest, giving priority to systematic reviews. Studies with repetitive information or of lesser relevance to the subject of this review were excluded. When appropriate, the PubMed database was also consulted to acquire useful information. Finally, 262 articles were selected.

We have deliberately tried to avoid technical terms in order to make this work as accessible as possible to all readers. Given the broad area of research analysed, description of the specific mechanisms of polyphenol activity in relation to health has been omitted. However, the extensive bibliography cited allows for in-depth exploration of the topics discussed hereafter.

## 3. Bibliometric Analysis from 1970 to 1999

**Figure 1:**
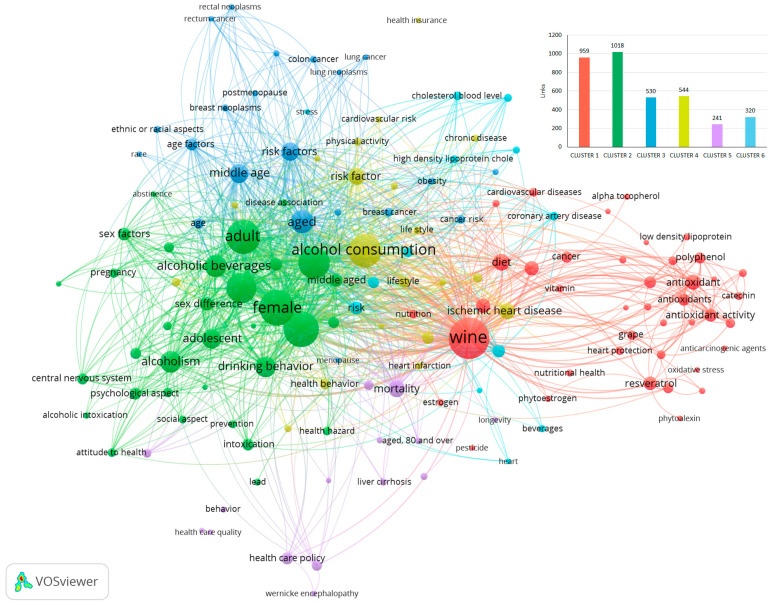
Co-occurrence analysis using VOSviewer (VOSviewer version 1.6.20) of terms in titles, abstracts, and keywords of documents obtained in Scopus from 1970 to 1999 using as keywords “Wine AND Health” (links for each cluster are reported in the bar-chart in the top right).

In Figure 1, it is possible to observe the bibliometric networks constructed by using the words “Wine AND Health” considering the period from 1970 to 1999. Obviously, the item “wine” was the most important one (cluster 1, 139 links, 231 occurrences, and a total link strength of 1646). It was followed by the items “alcohol consumption” (cluster 4, 129 links, 154 occurrences, and a total link strength of 1346), “alcohol” (cluster 2, 129 links, 140 occurrences, and a total link strength of 1237), “female” (cluster 2, 126 links, 176 occurrences, and a total link strength of 1588), “male” (cluster 2, 127 links, 174 occurrences, and a total link strength of 1528), “adult” (cluster 2, 113 links, 76 occurrences, and a total link strength of 1467), “aged” (cluster 3, 108 links, 48 occurrences, and a total link strength of 774), and “risk factor “ (cluster 3, 102 links, 52 occurrences, and a total link strength of 551).

In particular, six main clusters were created, with 143 items, 3612 links, and a total link strength of 13,984. Cluster 1, in red, mainly includes the items characterising wine and its health promoter molecules/effects (resveratrol, polyphenols, antioxidants, vitamins, health protection, etc.). Cluster 2, in green, contains those items linked to alcohol, alcoholism, age, gender, and psychological and social aspects. Cluster 3, in blue, mainly links age, race, risk factors, and various health diseases (breast cancer, colon cancer, diabetes mellitus, obesity, etc.). Cluster 4, in yellow, contains items like alcohol consumption, risk factors, and cardiovascular diseases. Clusters 5 and 6 are minor clusters. Specifically, cluster 5, in purple, includes items like mortality, ageing (80 and over), liver cirrhosis, and health care; cluster 6, in light blue, associates the items ethanol, blood pressure, cholesterol, coronary diseases, hypertension, risk, and stress.

This clearly shows that the period from 1970 to 1999 was greatly characterised by research involving (i) wine as a health promoter; (ii) alcohol abuse; (iii) wine and, in general, alcohol consumption and their impact on various diseases related to age, gender, and health status; and (iv) wine compounds with health effects. It should be noted that most of the studies conducted in the period from 1970 to 1999 refer to research concerning alcoholic beverages in general. We have therefore selected those papers referring primarily to wine, which will be discussed in the following paragraphs.

### 3.1. Wine as Health Promoter

In the 1970s, numerous studies were focused on wine’s microbiology and chemistry, with a special interest in the development of new methods for determining the molecules involved in wine quality, maturation, and ageing. Concurrently, alongside scientific work on the risks of alcohol consumption, data on the positive effects of wine consumption have also increased. One of the first sources reporting specific information on wine’s benefits for health was given by St. Leger et al. [8]. In their survey, the authors analysed mortality data in 18 different countries, considering variables like health services, economic and demographic factors, population density, cigarette consumption, alcohol consumption attributed separately to beer, wine, and spirits, calories, and fat intake. The principal finding was a strong and specific negative association between ischaemic heart disease deaths and wine consumption. Subsequently, Klurfeld and Kritchevsky [9] demonstrated the superiority of red wine over other alcoholic beverages in preventing atherosclerosis. Their observation was based on an animal study involving rabbits fed a high-cholesterol diet together with water (control) or different drinks containing ethanol. After 3 months, all control rabbits had developed atherosclerotic lesions in the coronary arteries. All alcoholic beverages except beer reduced the incidence of atherosclerotic lesions, but red wine showed the most impressive effect, lowering the incidence of lesions by 40%.

Baum-Baicker [10] reviewed the health benefits of moderate alcohol consumption, highlighting the inverse relationship between coronary heart disease (CHD) and regular alcoholic beverage use. This negative association was ascribed to alcohol’s effect of increasing levels of high-density lipoprotein cholesterol, which acted in the removal of cholesterol from tissue. Other explanations for alcohol’s moderate cardioprotective effect included the associated diet changes in moderate drinkers, the silicon content in wine and beer, decreased platelet aggregation and coagulation, and the ability to lessen stress and/or alter personality patterns associated with CHD risks. Dry, non-sweet wine and diluted, distilled spirits have been recommended in the treatment of diabetes. It has been suggested that alcohol may improve glucose tolerance and blood glucose response to ingested carbohydrates. Due to reported decreased HDL values in diabetics, alcohol has been suggested as useful for its HDL-increasing function. Authors have reported positive benefits of moderate wine consumption due to (i) trace metals and mineral content and (ii) a moderate cardioprotective effect due to alcohol increasing levels of high-density lipoprotein cholesterol. Later, Middleton [11] described the role of flavonoids from fruit, vegetables, wine, and tea in mammalian cellular functions and enzyme systems in relation to health and disease.

One of the most interesting publications on the effect of wine consumption on health is given by Renaud and de Lorgeril [12]. The authors analysed the data from the MONICA project, a worldwide monitoring system for coronary heart disease (CHD) organised by the World Health Organisation (WHO), and noted that the mortality rate for CHD in France was closer to that of Japan or China than that of the USA or the UK, particularly for women, although saturated fat intake and serum cholesterol concentrations were similar to those in the USA and the UK. This finding constituted the so-called French paradox. In particular, data from the city of Toulouse, France highlighted that wine should have a greater protective effect than other kinds of alcoholic beverages. The authors concluded that the protection from CHD was not only associated with low serum cholesterol or high HDL cholesterol due to alcohol consumption, as previously assumed by other authors, but also reduced platelet aggregation. Arguably, this publication set the point where wine, and no longer alcoholic beverages in general, was included, along with olive oil, fruit, and vegetables, among the key foods that characterise the Mediterranean diet, which differs from other European diets in its wealth of nutritious and non-nutritious antioxidants that may contribute to the persistence of health-protective attributes [13,14,15,16,17].

Of interest is the intervention study performed by Leighton et al. [18]. The authors reported the results of a 3-month survey on two groups of 21 male volunteers each who received either a Mediterranean diet or a high-fat diet. During the second month, red wine was added to the diets. The results obtained after clinical, physiological, and biochemical evaluations supported the following conclusions: (i) a high-fat diet induces oxidative stress; (ii) a diet rich in fruits and vegetables enhances antioxidant defences; and (iii) wine supplementation to a high-fat or Mediterranean diet increases plasma antioxidant capacity, decreases oxidative DNA damage, and normalises endothelial function.

### 3.2. Alcohol Abuse and Health

Another theme addressed in the period from 1970 1999 was alcohol abuse [19,20,21,22,23]. In addressing this topic, researchers referred to alcoholic beverages in general, not specifically wine. Many interventions have been developed by governments of various states with the intent of restraining heavy alcohol consumption. At the end of the 20th century, the main goals of alcohol-related policies in the United States were to diminish motor vehicle accidents and related injuries, lower the prevalence of alcoholism, and reduce health complications associated with alcohol abuse [24,25]. In different parts of the world, the type of alcoholic beverage differed on the basis of lifestyle, cultural characteristics, and policies, with liquors consumed especially in US populations, beer in Germany, and wine in other European countries [26]. Heavy alcohol consumption was associated with psychological effects, especially clinical depression [10], and health risk factors, highlighting the relationship between alcohol consumption and various diseases in cohorts of patients of different ages and genders. Marmot et al. [27] reported the results of a study on 1422 men, classified according to their average daily alcohol intake. Over 10 years of follow-up, the mortality rate was lower in men reporting moderate alcohol intake than in either non-drinkers or heavier drinkers. They found a U-shaped relationship between estimated daily alcohol consumption by age and mortality from all causes. Cardiovascular mortality was, however, greater in non-drinkers, and non-cardiovascular mortality was greater in heavier drinkers. As for other pathologies, a positive dose–response effect was largely evidenced, with alcohol designated as a direct and specific cause of various morbidities. Kune and Vitetta [28] reviewed the aetiology of colorectal cancer based on scientific evidences from 1957 to 1991, concluding that alcohol increases mucosal cell proliferation, activates intestinal procarcinogens, and has a role in unabsorbed carcinogens. In their review, the authors outlined that general or indirect carcinogenic effects of alcohol included immunodepression, activation of liver procarcinogens, changes in bile composition, and increased tissue nitrosamine levels.

Although the exact mechanisms through which chronic alcohol ingestion stimulates carcinogenesis was not known, experimental animal studies highlighted that ethanol is not a carcinogen, but, under certain conditions, it is a cocarcinogen and/or a tumour promoter [29]. Ethanol metabolism was also evaluated in relation to the generation of highly reactive compounds, such as acetaldehyde, unstable acetaldehyde–albumin complexes, and free radicals, able to bind to cell constituents, decrease DNA repair mechanisms, trap the detoxifying peptide glutathione, and induce chromosomal aberration. However, the amount of unstable cytotoxic complexes in the serum of healthy male volunteers was lower after drinking red wine than after drinking white wine, probably due to the higher concentration of antioxidants in red wine [30].

### 3.3. Wine and Its Impact on Various Diseases Related to Age and Gender

Among studies conducted in the period from 1970 to 1999, Leger et al. [8] assessed the factors associated with cardiac mortality in developed countries, highlighting that alcohol consumption, primarily wine, was very strongly negatively associated with ischaemic heart disease mortality and moderately positively associated with road accident deaths in both males and females separately screened in the 25–34 and 55–64 age groups. Williams et al. [31] conducted a large study on the association of cancer sites with tobacco and alcohol consumption. The researchers pointed out that alcoholic beverage intake was positively associated with a consistent dose–response increase in the risk of invasive cancers of the oral cavity and the larynx. Females also exhibited a positive association of alcohol with rectal cancer, whereas males did not. When age and race were controlled, a significant positive dose–response association was observed. In a similar study, Hoey et al. [32] suggested that alcohol and, particularly, red wine may be important risk factors for adenocarcinoma of the stomach in France. In addition, cigarette smoking, a risk factor in itself, when coupled with alcohol, appeared to markedly increase the risk. These findings were confirmed [33] or disproved [34,35] by other studies in the same field.

An important piece of research from the same period focused on alcohol consumption among women, with attention to the effect of drinking during pregnancy [36,37,38,39] and the possible relationship between wine consumption and breast cancer [40,41,42,43,44,45] or colorectal cancer [46,47,48,49].

### 3.4. Knowledge of Wine Compounds and Assessment of Health Effects in the Years 1970–1999

In light of the previous data, it is clear that until the end of the 20th century, the most discussed and controversial results concerned alcohol content in alcoholic beverages. However, much of the scientific world was overall in agreement regarding the inverse correlation between moderate alcohol consumption and the incidence of CHD. This was essentially ascribed to alcohol’s capacity to increase levels of serum high-density lipoprotein (HDL) cholesterol and to reduce platelet aggregation [12]. Moreover, alcohol was also designated as responsible for an acute effect on fibrinolysis, thus preventing the tendency to increased thrombus formation [50]. In work by Obisesan et al. [51], the authors hypothesised that alcohol consumption was a risk factor for the development of age-related macular degeneration (AMD). The deleterious effect of alcohol on hypertension and its adverse effect on some forms of retinopathy formed the basis for this hypothesis. Surprisingly, the study showed a negative association between alcohol consumption and the odds of developing AMD. Red wine was reported as the main actor in this negative association.

In wine, ethanol is the most abundant alcohol. In standard fermentation conditions, ethanol can accumulate to about 14–15%, even if its concentration generally ranges between 10 and 13% [52]. Methanol is also present in wine at minor concentrations (about 0.1–0.2 g/L). Because it is predominantly generated from the enzymatic breakdown of pectins, pectolytic enzymes added to support wine clarification could accidentally increase the methanol content. Methanol is neurotoxic, and its metabolites, formaldehyde and formic acid, can cause organic lesions and central respiratory system disorders. It must, however, be considered that wine has lower concentrations of methanol and its metabolites in comparison to other alcoholic or non-alcoholic beverages [53,54]. In addition to the health effects of alcohol, researchers in the years 1970–1999 also extensively evaluated the effect of polyphenolic compounds, particularly anthocyanins, tannins, and flavonoids. These compounds had already been known in the wine industry for some time, but they were considered mainly for their contribution to wine’s characteristics, such as colour, sensory effects, aroma, and texture. On the other hand, polyphenols from various vegetables have already been indicated for their antioxidant activity [55,56]. However, it was not until the beginning of 1990 that wine polyphenols were definitely recognised, together with moderate alcohol intake, as health promoters. The number of compounds identified in wine increased over time (see Table 1) thanks to the development of gas chromatography, high-pressure liquid chromatography, nuclear magnetic resonance, thin-layer chromatography, and infrared spectroscopy [57].

Among phenolic compounds, wine flavonoids were recognised as potent antioxidants and metal chelators. Their capacity to sequester and thus reduce the activity of oxidant-inducing metals, such as iron and copper, was attributed to their 4-carbonyl and 1,5- (or 3-) hydroxyl groups [52]. Another class of compounds examined in the same period was stilbenes and stilbene glycosides. Among them, the most extensively studied was *trans*-resveratrol (3,5,4’-trihydroxystilbene). This compound was shown to modulate lipid metabolism and inhibit low-density lipoprotein oxidation and platelet aggregation. Furthermore, as a phytoestrogen, resveratrol could provide cardiovascular protection. Resveratrol has also been claimed for its anti-inflammatory and anti-cancer properties. [52,58,59,60,61].

Other phenolics extensively studied for their antioxidant potential were the hydroxycinnamic acids (caffeic, ferulic, and *p*-coumaric acids), catechins, and epicatechin [62,63,64]. The universal property highlighted for all of these compounds was related to their function as antioxidants, expressed by their ability to capture free radicals and inhibit their enzymatic generation and to inhibit the oxidation of cellular components, such as membranes and low-density lipoprotein. Studies from those years showed that some of these polyphenols were able to prevent or reduce platelet aggregation, the synthesis of pro-aggregative eicosanoids, and synthesis by leukocytes of pro-inflammatory leukotrienes. In addition, certain polyphenols were demonstrated to be able to promote prostacyclin and nitric oxide synthesis and thus play a role in optimising blood flow through the arterial system. Finally, it was shown that quercetin in particular had anti-cancer potential, while other phenols may have a weak lipid-lowering capacity [52].

**Figure 2:**
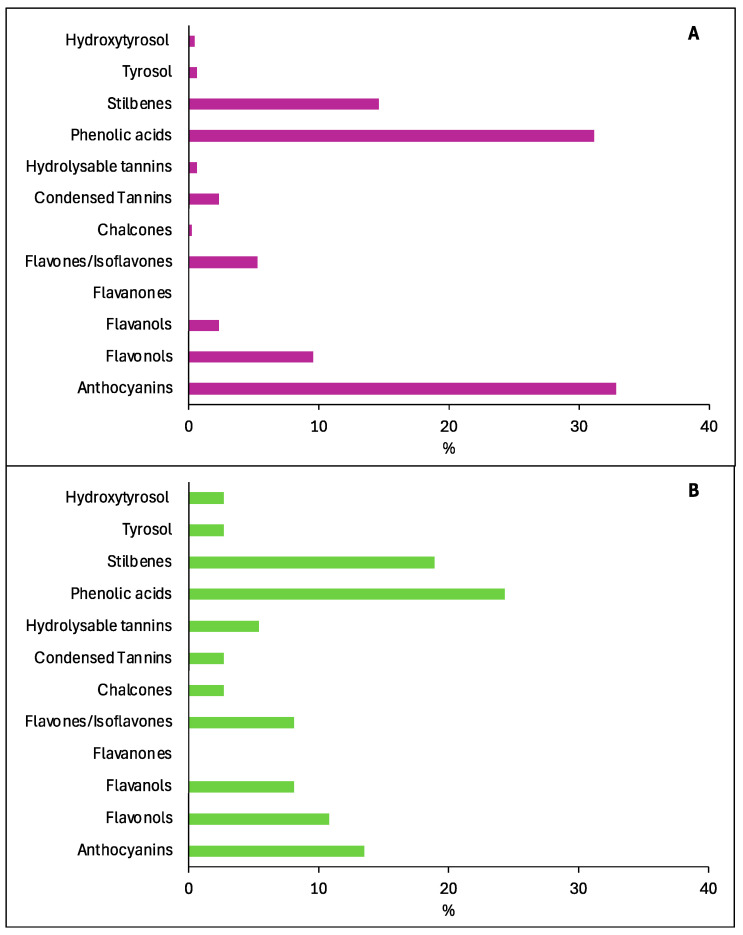
Amount of documents (%) obtained in Scopus from 1970 to 1999 using as keywords “Wine AND name of a single class of compounds” (**A**) and “Wine AND name of a single class of compounds AND Health” (**B**).

Figure 2 may be useful to recognise the most studied phenolic compounds in wines in the years 1970–1999. In particular, many of the detected compounds belonged to phenolic acids and anthocyanins, followed by stilbenes, flavonols, flavones/isoflavones, flavanols, and condensed tannins. Few other compounds belonging to the remaining classes were reported (Figure 2A). Considering the studies concerning the health effects of these phenols, data from the literature indicate phenolic acids and stilbenes as the most indagated wine-derived compounds, followed by anthocyanins, flavonols, flavanols, flavones/isoflavones, and hydrolysable tannins (Figure 2B). All other classes of compounds have been little investigated for their health effects.

The presence of polyphenols was gradually correlated with red wine, which proved its superiority over other alcoholic beverages, including white wine, in terms of type and concentration of active compounds and, consequently, health activity [65,66].

**Table 1:** Phenolic compounds detected in wine and tested for their health effects in the years 1970–1999 (the references are the main documents describing the detection of a compound in wine and the evaluation of health effects).

Compound	Detection in Wine	Evaluation of Health Effects
FLAVONOIDS		
Anthocyanins		
Delphinidin	[64,67,68]	[63,69,70]
Cyanidin		
Petunidin		
Peonidin		
Mavidin		
Vitisins	[71,72]	
Others	[72,73,74]	
**Flavonols**		
Myricetin	[64,75]	[64,75]
Quercetin	[64,75]	
Kaempferol	[75]	[75]
Isorhamnetin	[75]	
Rutin	[75]	
**Flavanols**		
Catechin	[63,64]	[63]
Epicatechin	[63,64]	[63]
Epigallocatechin	[76]	[76]
Epicatechin-gallate	[76]	[76]
Astilbin	[77]	[78]
Engeletin	[77]	[76]
**Flavanones**		
**Flavones/isoflavones**	[79]	[80]
**Chalcones**	[79]	[81]
**Condensed tannins**	[79,82,83]	[52]
**Hydrolysable tannins**		
Gallotannins	[84]	[52,85]
Ellagitannins	[62,86]	[52,85]
**Other phenols**	[87]	
**NON-FLAVONOIDS**		
**Phenolic acids**		
***Hydroxybenzoic acids***		
*p*-hydroxybenzoic acid	[62,68,88]	[89]
Gallic acid	[62]	[63]
Vanillic acid	[62,86]	[90]
Gentisic acid	[91]	[92]
Syringic acid	[62,86]	
Salicylic acid	[68]	[52]
Protocatechuic acid	[93]	[93]
***Hydroxycinnamic acids***		
Caffeic acid	[62,63,64]	[63]
Coumaric acid	[62,67]	[94]
Sinapic acid	[95]	[95]
Ferulic acid	[62]	[96]
**Stilbenes**		
*trans*-resveratrol	[97,98]	[52,58,59,60,61]
*trans*-piceid	[98,99]	
*trans*-astringin	[99,100]	
Piceatannol	[99]	
**Tyrosol**	[68]	[101]
**Hydroxytyrosol**	[68]	[94]

A more detailed description of the health effects of individual classes of phenolic compounds is given in the following paragraphs.

## 4. Bibliometric Analysis from 2000 to 2024

**Figure 3:**
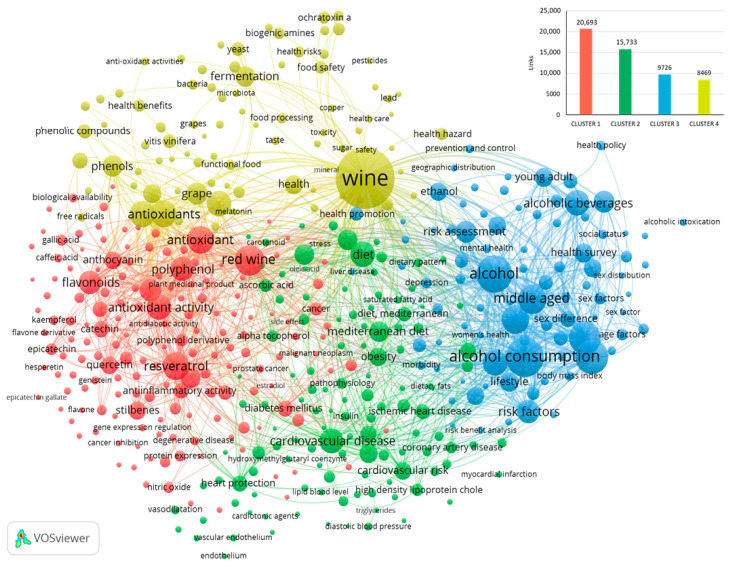
Co-occurrence analysis using VOSviewer (VOSviewer version 1.6.20) of terms in titles, abstracts, and keywords of documents obtained in Scopus from 2000 to 2024 using as keywords “Wine AND Health” (links for each cluster are reported in the bar-chart at the top right).

Figure 3 shows the bibliometric networks constructed by using the words “Wine AND Health” in the subsequent period from 2000 to 2024. The item “wine” was the most important (cluster 4, 455 links, 2575 occurrences, and a total link strength of 22,035), as the research was set up using the keywords “wine” AND “health”. This item was followed again, as already observed in the previous years, 1970–1999 (Figure 1), by “alcohol consumption” (cluster 3, 436 links, 1070 occurrences, and a total link strength of 12,400) and “alcohol” (cluster 3, 443 links, 939 occurrences, and a total link strength of 10,207). The items “red wine” (cluster 1, 452 links, 661 occurrences, and a total link strength of 9199), “resveratrol” (cluster 1, 433 links, 535 occurrences, and a total link strength of 7332), and “antioxidants” (cluster 4, 421 links, 508 occurrences, and a total link strength of 6446) followed.

In detail, there were four main clusters, 456 items, 54,621 links, and a total link strength of 298,550. Cluster 1, in red, mainly includes the items characterising red wine and its health promoter molecules/effects (resveratrol, polyphenols, antioxidants, flavonoids, antidiabetic activity, anti-inflammatory activity, cancer inhibition, degenerative disease, etc.). Cluster 2, in green, contains diet, including Mediterranean diet, and various diet-related problems (obesity, cardiovascular disease/risk, blood pressure, triglycerides, uric acids, etc). Cluster 3, in blue, comprises many items containing the word “alcohol” (alcohol consumption, alcohol abuse, alcohol drinking, alcohol intoxication, alcoholic beverages, etc.) and problems related to alcohol consumption (anxiety, breast cancer, cancer incidence, cancer mortality, liver cirrhosis, mortality risk, etc.). In this cluster are also included the items related to age, gender, race, and socio-economic factors. Cluster 4, in yellow, contains the item “wine”, a miscellanea of other items found in clusters 1–3, and items related to mycotoxins, microorganisms, and metals.

Through data analysis of Figure 1 and Figure 3, it is evident that in the period 2000–2024, much higher links, occurrences, and total link strength were found compared to the previous period of 1970–1999, reflecting an expansion of research concerning wine over the last 24 years. The field of research regarding alcohol consumption as a risk factor linked to age, gender, race, and socio-economic aspects remained highly monitored. Most of all, red wine and antioxidants, particularly resveratrol, assumed particular importance over the years.

### 4.1. Awareness of the Health Implications of Wine Consumption in the Years 2000–2024

In the early 2000s, thanks to numerous epidemiological surveys, it was generally recognised that moderate alcohol intake, particularly wine, was associated with a lower risk of cardiovascular disease [102]. It was also clear that these benefits could be enhanced by red wine consumption due to the additional effects of polyphenols, which demonstrated beneficial properties independent from the presence of alcohol [102]. Evidence that moderate and regular red wine consumption may prevent the onset of type 2 diabetes and related complications was furnished [103]. Epidemiological studies showed that moderate intake of red wine was able to reduce the risk of developing neurological disorders [104], especially when Alzheimer’s and Parkinson’s diseases were considered [105]. The capability of red wine to protect against various immune-related disorders by stimulating the immune response and by reducing inflammation was reviewed [106]. Guilford and Pezzuto [107] highlighted that both the alcoholic and polyphenolic components of wine can contribute to its beneficial effects in healthy people, given wine is a complex mixture in which a multitude of chemical constituents and metabolites work synergistically to impact human health. Clinical studies and research have continued over the years indicating beneficial effects resulting from moderate wine, especially red wine, consumption [108,109,110], particularly when included in a Mediterranean diet model [111,112,113].

While studies on the positive effects of wine have multiplied, attention to possible health risks has remained high, and scientists continue to warn consumers about the harmful effects of heavy alcohol consumption. In a cohort study based on information obtained over 6 years of follow-up, Kao et al. [114] reported that even if moderate alcohol (wine, beer, or spirits) consumption does not increase the risk of type 2 diabetes in either middle-aged men or women, high alcohol intake increased diabetes risk among middle-aged men. When wine consumers were examined, no statistically significant association was found between wine intake and diabetes risk. Remarkably, the authors did not associate this finding with the presence of health-promoting polyphenols in wine but with healthier behaviours (increased physical activity, total dietary fibre intake, and decreased total fat intake) of wine-drinking subjects.

In the case of alcoholic cirrhosis, Pelletier et al. [115] pointed out that although wine contains several polyphenolic compounds with antioxidant properties that may alleviate ethanol-induced liver toxicity, its heavy consumption can still lead to alcoholic cirrhosis, like other alcoholic beverages. Other studies indicated that in populations with frequent wine consumption, the risk of cancer of the oral cavity and pharynx was strongly increased [116]. The authors emphasised the carcinogenic effect of alcohol, underlining that wine contains a concentration of resveratrol that is probably insufficient to promote a biological effect and counteract the deleterious impact of alcohol.

Chronic alcohol intake was associated with increased breast cancer risk in women [117], even if data from the literature highlighted the cancer-preventing effects of resveratrol from wine [107,118,119]. Other negative effects of wine intake regarded induced asthmatic symptoms attributable to natural sulphite levels or added sulphur dioxide, the presence of the carcinogen ethyl carbamate derived from arginine metabolism in malolactic bacteria [120], and possible toxic effects of heavy metals [121] or mycotoxins [122].

### 4.2. Assessment of Health Effects of New and Already Known Wine Compounds

From the data shown in Table 1, it is evident that most of the wine compounds known today had already been identified and studied for their health effects before 2000. New wine phenols were detected in the subsequent period (Table 2), but they were generally derived through processes of rearrangement of the original phenols detected previously, mainly due to wine making and ageing processes or interaction with microbial metabolites.

#### 4.2.1. Wine Anthocyanins

Among flavonoids, already known anthocyanins (Table 1), as well as their new derivates (Table 2), have received attention not only for their important role in wine colour and sensory attributes but also for their potent antioxidant and anti-inflammatory effects [123,124,125,126,127,128,129]. Initially, anthocyanin derivatives were assumed to derive mainly from direct or acetaldehyde-mediated reactions of condensation between anthocyanins and flavanols. Over the years, reactions involving anthocyanins with other compounds, such as pyruvic acid, vinylphenol, vinylcatechol, ketoglutaric acid, acetone, and 4-vinylguaiacol, have been demonstrated, giving rise to new anthocyanin-derived pigments called pyranoanthocyanins, including pinotin A and vitisins [130]. Overall, these new pigments showed even higher antiradical and antioxidant activity than their precursors thanks to the inclusion in their structure of a catechol or catechin moiety able to donate a H atom to free radicals, thus inactivating them [130,131].

The absorption and bioavailability of catechins in the human body, together with their microbial catabolism, were clarified, and numerous in vitro and in vivo studies have proven a wide range of beneficial effects on human health, so much so that a daily dose of anthocyanins of 50 mg was recommended in some countries [132]. Today, we know that anthocyanins are absorbed and metabolised throughout the entire gastrointestinal tract, resulting in the production of many different intermediate metabolites that possess specific biological properties and activities, such as endothelial cell activation and repair, improvement of cognition and neuroprotection thanks to the ability to decrease brain oxidative stress, inflammation, and degeneration [128]. Particularly in the colon, anthocyanins and their metabolites are further broken down into phenolic acids, which can be easily absorbed by the intestinal mucosa. These products can modulate the colonic microbiota, resulting in increased production of short-chain fatty acids (SCFA), known to be involved in regulating the functions of the gut–microbiota–brain axis [133]. Interestingly, in 2011, wine was indicated for its contribution (up to 25%) of dietary anthocyanin intake in the European population [134].

#### 4.2.2. Wine Flavonols

With regard to flavonols, syringetin and laricitrin were first detected in 2003 and 2007, respectively, in Cabernet Sauvignon wine during ageing [135] and in red grapes and wines [136]. A recent review by Chmiel and Stompor-Gorący [137] summarises the current reports on syringetin, a dimethyl myricetin derivative, and some of its structural analogues already known for their antioxidant, antimutagenic, hepatoprotective, antidiabetic, and antilipogenic properties. Specifically, these methylated forms of flavonols showed high stability, biological activity, and an ability to interact with cell membranes, with the resulting ability to inhibit cancer cell proliferation. Syringetin was also suggested as an optimal adjuvant in the treatment of diabetes thanks to its ability to inhibit the absorption of carbohydrates and reduce postprandial glycemia [138], its status as a modulator of the accumulation of age-related pigments with a strong pro-longevity effect [139], its anti-viral effects [140], its benefits for the treatment of Alzheimer’s disease [141,142], and its status as a stimulant of osteoblast differentiation at various stages [143]. Most of these effects were also highlighted for laricitrin and for the already known flavonol isorhamnetin [137].

#### 4.2.3. Wine Flavanols

Evidence regarding the health benefits of dietary flavanols has continued to accumulate over the years. In particular, their effect on cardiovascular health has been extensively evaluated, with procyanidins detected as the principal vasoactive polyphenols in red wine [144]. Interestingly, Corder et al. [144] showed a high correlation between longevity and high content of oligomeric procyanidins in red wines from southwest France due to traditional winemaking, outlining the positive effects of these compounds on vascular functions. Similar results have been reported more recently by Khan et al. [145], who strongly indicated that oligomeric procyanidins play a key role in the improvements in endothelial function caused by red wine. The mechanisms underlying the anti-inflammatory effects of procyanidins were already described by Martinez-Micaelo et al. [146], who emphasised the involvement, among others, of the modulation of several pivotal pathways in the regulation of cellular homeostasis, the production and secretion of inflammatory mediators (e.g., cytokines or nitric oxide), and the modulation of mitogen-activated protein kinase and nuclear factor κB pathways, involved in the regulation of several cellular processes.

Among flavanols, the already known astilbin (see Table 1) was indicated as a possible agent in the prevention and management of various diseases and disorders [147]. Its mechanism of action was clarified in relation to the central nervous system and the immune system by acting on various immunomodulators and inflammatory modulators. Many other properties (anti-inflammatory and antimicrobial activity, hepatoprotective and antioxidative action) and the proposed mechanisms of this phenol were also elucidated.

#### 4.2.4. Wine Flavanones

Among new compounds, Jandera et al. [148] described the presence in red wines of naringenin and hesperidin using RP-HPLC analysis with a CoulArray detector. The occurrence of these flavanones was already reported almost exclusively in citrus fruits, even if low amounts of naringenin was also detected in tomatoes and tomato-based products [149]. Their antimicrobial [150,151] and health benefits [152] were detected. In rat models these flavanones were more efficiently absorbed than other flavonoids, thus suggesting a dietary role in reducing the risk of chronic diseases, such as cardiovascular diseases and cancer, despite their limited amount in wine.

#### 4.2.5. Wine Phenolic Acids

To our knowledge, only a few other phenolic acids have been added to those already known by 1999 and listed in Table 1. The impact of these compounds on health has been continually studied, as they are an important fraction of wine polyphenols.

Their antioxidant activity was determined in vitro [153], while their vasodilatory activity had already been recognised a few years earlier [154]. Especially caffeic and ferulic acids, the dominant phenolic acids in wines, were evaluated for their important role as anticancer agents [155,156]. Behind these activities, today, we know that phenolic acid metabolites, as well as those from other polyphenols, are mainly formed from the metabolism of gut microbiota, which, in addition to their own antioxidant activities [157], could be responsible for much of the disease reduction associated with wine consumption [158]. For this reason, in vitro studies could give rise to very different results with respect to those actually obtained in vivo [159]. As with the other phenols, recent reviews on the health effects of the main phenolic acids are available in the literature, confirming their importance in the human diet [110,160,161].

#### 4.2.6. Wine Stilbenes

Monomeric stilbenes were extensively studied for their health properties [123,162,163,164,165,166,167,168] and antimicrobial activity [169]. In 2013, Moss et al. [170] highlighted for the first time the presence in red wine samples of dimeric, trimeric, and tetrameric forms (Table 2) derived from previously identified stilbene monomers (see Table 1). In particular, 23 new compounds were found, including several resveratrol–resveratrol and piceatannol–piceatannol homodimers, resveratrol–piceatannol heterodimers, modified dimers (*O*-glycosylated, methoxylated, and oxidised) and multiple trimers and tetramers. The study emphasised the chemical complexity of stilbenes in wine, which is the most important source of these compounds in the Western diet [171]. In fact, stilbene intake from wine accounts for 98.4% of intake, followed by 1.6% represented by grape berries and grape juice [172].

El Khawand et al. [171] reviewed the biological activity of monomer and oligomer stilbenes. It was observed that stilbene monomers present a higher volume of distribution than stilbene oligomers, suggesting that monomers are better distributed from blood to tissues than oligomers in accordance with the higher absorption rate of monomers in comparison with oligomers. This fact would suggest that non-absorbed or low-absorbed oligomers could exert local action, primarily in the intestine, whereas monomers could exert a systemic, beneficial action upon absorption and delivery to target tissues.

Despite the wide range of wine stilbenes with health effects and the multiple mechanisms of action of each compound, resveratrol remained strongly addressed and extensively studied. Its beneficial effects on health have been widely examined over the years [66,108,109,111,173,174,175,176,177,178,179,180,181,182,183]. Apart from the already recognised antioxidant activity, researchers have reported resveratrol as a potent antiarrhythmic agent with cardioprotective properties protective against cerebral ischemic injury thanks to its capacity to cross the blood–brain barrier and metabolic diseases by improving mitochondrial function; it is also cancer chemo-preventive. A few studies reviewed potential adverse effects of resveratrol [184,185], but they were primarily related to possible toxic effects of resveratrol supplementation in humans when administered in the form of a pharmaceutical drug.

Finally, it should be emphasised that despite the numerous positive health effects that resveratrol has been shown to possess, its content in wine is very low [158,186], so it cannot be considered the preferred source of supply of this compound. Because of the important properties of resveratrol, Bavaresco et al. [187] reviewed the possibility of producing high-resveratrol-containing grapes and wines through the application of winemaking technologies that avoid the degradation of this compound and its oligomers, viniferins, including viticultural factors, grape variety, precautions during vinification and storage, and yeast choice.

#### 4.2.7. Wine Hydrolysable Tannins

Gallotannins and ellagitannins were already known for their influence on the ageing of wine in oak barrels (wood). While they were extensively explored for the influence on the organoleptic properties of wines [188,189,190,191], these wine compounds have been little studied for their health benefits, probably due to their low content, as most consumed wine is not aged in oak barrels and, even when aged there, their content in wines depends on the barrel used and the duration of ageing [192].

Over the years, the health effects of hydrolysable tannins from various sources have been studied, but we have been unable to obtain information on wine-specific compounds. In 1989, Okuda et al. [193] reported the antitumor activity exhibited by several oligomeric ellagitannins and their anti-HIV effects. Other biological activities, such as the inhibition of the mutagenicity of carcinogens and the inhibition of tumour promotion, were also reported. In 2000, Clifford and Scalbert [194] examined the dietary effect on health of ellagitannins consumed with foods and beverages, including wine. Some ellagitannin derivates were described by Quideau et al. [195]. The authors reported that some of these compounds expressed pharmacologically relevant activities, suggesting a potential antiproliferative action and their potential use as new anticancer drugs. Later, Okuda and Ito [196] reported biological and pharmacological activities of tannins, including ellagitannins. They described host-mediated antitumor activities and antimicrobial activities against *Helicobacter pylori*, antibiotic-resistant bacteria, and *Leishmania donovani*. More recently [197,198], various types of urolithins derived from the intestinal microbial degradation of ellagitannins have attracted the attention of scientists for their antioxidant and anticarcinogenic effects. Furthermore, the antiproliferative and apoptosis-inducing activities of urolithins have been demonstrated.

**Table 2:** New phenolic compounds detected in wine and tested for their health effects in the years 2000–2024 (the references are the main documents describing the detection of a compound in wine and the evaluation of its health effects).

Compound	Detection in Wine	Evaluation of Health Effects
FLAVONOIDS		
Anthocyanins		
Anthocyanin derivates	[199,200,201]	[123,124,125,126,127,128,129,130,131,202,203]
Pinotin A	[204]	
Oxovitisins	[205]	
Others	[206,207,208]	
**Flavonols**		[209]
Laricitrin	[136]	[210]
Syringetin	[135]	[137]
**Flavanols**		
Flavanol monomer and dimer hexosides	[211]	[144,145]
Flavanol derivates	[201]	
**Flavanones**		
Naringenin	[148]	[152]
**Flavones/isoflavones**		[212]
**Chalcones**		[213]
**Condensed tannins**		[146]
**Hydrolysable tannins**		
Ellagitannin derivates	[195]	[195]
**Other flavonoids**	[214]	
**NON-FLAVONOIDS**		
**Phenolic acids**		
Various hydroxy acids	[215]	[110]
**Stilbenes**		
Hopeaphenol	[216]	[217]
*C*-Glucosides of resveratrol	[218]	[219]
Parthenocessin A	[220]	
Pallidol	[218]	[217]
Viniferins	[221]	[217]
Miyabenol C	[170]	[222]
Ampelopsin D	[170]	[223]
*trans*-ω-viniferin	[170]	[224]
*cis*-scirpusin A	[170]	
*trans*-scirpusin A	[170]	
Restrisol A	[170]	
Parthenostilbenin A	[170]	
Parthenostilbenin B	[170]	
(*E*)-miyabenol C	[170]	[217]
(*Z*)-miyabenol C	[170]	[217]
Other stilbenoids	[170]	
**Other non-flavonoids**	[214]	

**Figure 4:**
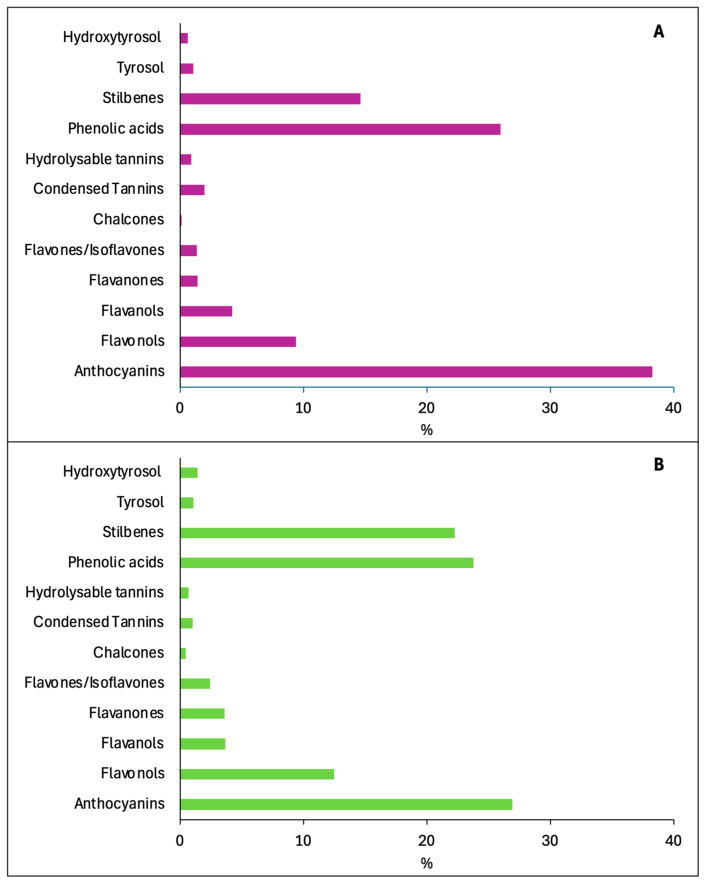
Amount of documents (%) obtained in Scopus from 2000 to 2024 using as keywords “Wine AND name of a single class of compounds” (**A**) and “Wine AND name of a single class of compounds AND Health” (**B**).

As illustrated in Figure 4, the interest of researchers in the study of new or already known phenolic compounds in wine has remained high from 2000 onwards. Interestingly, Figure 4A appears similar to Figure 2A, with the same classes of compounds being studied more than others for their presence in wine. When their health activity was considered (Figure 4B), we found some differences in comparison to the period from 1970 to 1999 (Figure 2B). In fact, anthocyanins received more attention than in the previous period, while stilbenes, phenolic acids, flavonols, and flavanols attracted about the same interest from scientists. For all other phenolics classes, fewer publications were recorded than for the previous classes.

### 4.3. New Insights into the Role of Wine in Human Health

From the evidence described above, it is quite evident that moderate consumption of wine, especially red wine, is associated with healthy properties due mainly to the strong antioxidant properties of phenolic compounds. It is also known that excessive alcohol consumption increases the risk of several pathologies, such as liver cirrhosis and various types of cancers. Moreover, other important considerations regarding the actual health benefits of wine consumption have emerged over the years, which are mainly related to the lack of epidemiological and in vivo studies, the presence of a large number of variables, such as diet, consumer age, gender differences, metabolism of phenolic compounds, degradation and transformation processes by the gut microbiota, and, last but not least, the complex chemistry of wine [127,225]. Scientists have highlighted the need to establish a cause and effect relationship between specific wine polyphenols and the reduction in specific diseases [145,226]. This implies the recognition of plausible mechanisms of action, the presence in wine of adequate polyphenol content to confer the proposed effect, the real absorption and bioavailability of active compounds, and evidence of a dose–response relationship. However, most of the health benefits of phenols, especially considering the past 24 years, refer mainly to single purified compounds, which are similar or analogous to those in wine but sometimes isolated from other sources and tested in experimental or animal models but not in human studies. In this way, the concept of wine as a complex mixture in which a multitude of chemical constituents and metabolites work synergistically to impact human health is completely lost.

Considering the vast amount of research over the past 24 years, as already mentioned, only a limited number of studies have reported human interventions to study the effect of wine’s ingestion on health [109,227,228,229,230,231,232]. In almost all scientific articles, moderate, light, or low alcohol consumption is suggested, but the actual amount corresponding to the above definitions cannot be clearly stated considering the numerous variables depending, on the one hand, on consumer features and, on the other hand, on the beverage ingested. Aspects like age, gender, ethnicity, genetics, health status, type of alcoholic beverage, frequency of consumption, diet, and other factors determine individual variability in the effects of alcohol intake on health. According to the WHO and the US National Institute on Alcohol Abuse and Alcoholism, the measure most frequently used is a “standard drink”, corresponding to the amount of alcohol an average adult can metabolise in 1 h [233]. In the UK, a standard drink is defined as an alcoholic beverage that contains 8 g of pure alcohol (10 mL). In other countries, a standard drink corresponds to 10 g of pure alcohol (12.5 mL), and, in the United States, this amount increases to 14 g of pure alcohol (17.5 mL) [234].

Considering wine’s features, the alcohol content can vary considerably from 8% to over 15%, and the total polyphenolic content can vary significantly from approx. 0.2 to 5 g/L, as well [235]. Furthermore, the amount, types, properties, and effects of each phenolic compound are strongly influenced by a myriad of factors, such as grape variety, climatic conditions, winemaking process, ageing, yeasts and bacteria, additives, etc. Finally, it is now clear that intestinal microorganisms play an essential role in the metabolism and absorption of certain phenols due to the production of new metabolites, some with greater and others with lesser health benefits than the original compounds [127]. For all of these reasons, the most frequent conclusion of past and present research is the need for further investigation to address all issues concerning wine and health.

## 5. Low-Alcohol and Dealcoholised Wine

As it is now well-known, the EU regulation 2021/2117 recently introduced the possibility to produce low-alcohol and alcohol-free wines. The directive defines “partially dealcoholized” wine as wines with actual alcoholic strength above 0.5% by volume and below the minimum actual alcoholic strength of the category before dealcoholisation, and “dealcoholized” wines include wines where the actual alcoholic strength is no more than 0.5% by volume. In other countries, the alcohol content of no- or low-alcoholic wines could be different. For instance, in the United States the label “low-alcohol wine” is used for products with alcohol content less than 8.5% by volume, and “alcohol free”, “non-alcoholic”, or “dealcoholized” are applied to wines with alcohol content below 0.5%. In Australia, the category “low-alcohol wine” indicates wines with alcohol ranging between 1.15 and 0.5% by volume, while alcohol content below 0.5% by volume is generally adopted for “alcohol-free”, “non-alcoholic”, or “dealcoholized wines”. The UK classifies as “low-alcohol wine” those wines with alcohol below 1.2% by volume and “alcohol-free” as wines with alcohol below 0.05% by volume [7].

In the following section, we will briefly discuss the possible health implications of these new types of wine. The dealcoholisation processes, which have already been examined by other authors [236,237,238,239], are also briefly reported, considering their impact on both consumer health and the sensory quality of wines.

### 5.1. Low-Alcohol and Dealcoholised Wine Market

Changing consumer preferences are accounted for as the main factors influencing the emergence of new wine products with low or no alcohol content [240]. However, other reasons have to be considered, which are mainly related to inflation and the economic downturn, which have progressively led to a reduction in wine consumption, as indicated by market data [241]. The decline in the world wine trade in recent years is due to a mix of factors. For instance, geopolitical tensions, such as the Russian–Ukrainian conflict or the most recent tariff war announced by the United States, create an uncertain global context and are eroding purchasing power. These conditions, together with slow economic growth, contribute to the decline in overall purchasing power and the consequent change in consumer preferences. As for wine and, in general, alcoholic beverages, different initiatives have been undertaken to reduce the harmful use of alcohol and to make consumers aware of the deleterious effects of alcohol intake [242,243], strongly conditioning the wine market. In this context, wine is at a competitive disadvantage, and wine companies need to adapt their investments to better align with new consumer choices (in economic and health terms). Trends clearly show that innovative alcoholic beverages, such as high-quality beers and low- and no-alcohol wines, are able to attract young consumers, together with lighter and less expensive wines, such as some white and sparkling wines [241,244].

### 5.2. Dealcoholisation Techniques: Impact on Consumer Health and Sensory Quality of Wines

A partial or total reduction in alcohol content can be obtained throughout the various stages of wine production (pre-fermentative, fermentative, and post-fermentation phases) using different approaches. Viticultural practices, such as decreasing the ratio between leaf area and fruit weight, irrigation procedures, and early harvesting, are pre-fermentative practices that have been shown to reduce the concentration of sugar in the berries [7]. Other pre-fermentative approaches include the dilution or membrane filtration of juice and the addition of glucose oxidase in must. Microbiological strategies, such as selecting and using yeasts that have low efficiency in converting sugars to alcohol, represent fermentation approaches that lead to the production of low-alcohol wines [241]. Post-fermentation technologies, which involve the physical dealcoholisation (the removal or reduction of ethanol) of wine, include membrane-based technologies (nanofiltration, reverse osmosis, pervaporation, osmotic distillation, and multi-stage membrane systems) and vacuum distillation procedures (vacuum distillation, spinning cone column). Other techniques comprise supercritical liquid extraction, stripping, and freeze concentration [245]. All of the above dealcoholisation techniques do not have a direct impact on consumer health, as none of them involve the use of chemicals added to the grapes, must, or wine at various stages. However, post-fermentative techniques modify to various degrees the chemical composition of dealcoholised wines, with possible health implications. For instance, partial or complete alcohol removal may facilitate the oxidisation of phenols, organic acids, and other volatile compounds, leading to the formation of reactive compounds, such as acetaldehyde [246], the amount of which must be evaluated in relation to possible health implications. On the other hand, the reduction in alcohol volume leads to an increase in key bioactive compounds by 2.5 to 3 times, such as anthocyanins and resveratrol, with positive effects on the health of consumers [237].

Changing the chemical composition of wines with no or low alcohol content leads to another key problem: the potential imbalance in flavour and aroma. In fact, the various dealcoholisation techniques alter, with different degrees of intensity, the composition in phenolic and volatile organic compounds, affecting the overall chemical and sensory profile of the wine. Specifically, the greater the degree of dealcoholisation, the greater the loss of sensory attributes of the wine. Kumar et al. [7] reported that a substantial amount of phenolics, volatile compounds, and sensory attributes were retained in wines after removal of ethanol content between 1 and 4% *v*/*v*. Furthermore, dealcoholised wine with ethanol content below 3% *v/v* ethanol showed more than 90% loss in volatile compounds and was poor in terms of sensory quality.

### 5.3. Health Benefits of the Consumption of Low- and No-Alcohol Wines

Alcoholic beverages, including wine, are consumed by different world populations, with considerable variations between countries. The large body of research reported here, covering about five decades, has shown that moderate alcohol consumption can have a cardioprotective effect and beneficial activity in other heart diseases, ischaemic stroke, diabetes mellitus, and, in general, death from all causes. Additional health benefits derive from the phenolic compounds contained in wine, particularly red wine, and studies on regular, moderate consumption of wine with food have shown both short-term and long-term positive effects, especially in individuals consuming a well-balanced diet [247]. Moderate wine consumption can have other positive effects, such as socialising and creating an optimistic or stress-free state of mind [248]. On the other hand, other authors argue that even moderate alcohol consumption can lead to harmful health consequences [249,250], and the report from the World Health Organization [251] highlights that 2.6 million deaths in 2019 were attributable to alcohol consumption, including deaths from cardiovascular diseases, cancer, injuries, such as those from traffic crashes, self-harm, interpersonal violence, and deaths linked to increased risk of sexually transmitted diseases. As expected, the overall association of wine consumption with health is complex, and the health risks associated with moderate alcohol consumption continue to be a controversial and complicated issue [252].

### 5.4. Consideration of Health Implications of Low- and No-Alcohol Wine Consumption

#### 5.4.1. Cardioprotective Benefits of Low- and No-Alcohol Wine

The EU Regulation 1308/2013 defined “Wine” as the product obtained exclusively from the total or partial alcoholic fermentation of fresh grapes, whether or not crushed, or of grape must. Wine has an actual alcoholic strength of not less than 8.5%. This amount can slightly vary in other countries, but alcohol content in wine is generally above 7.5%. Consequently, scientific research on the effects of wine consumption on health has so far involved wine as a whole. Little is yet known about the consumption of dealcoholised wine. Considering that more than 50 years later, the effect of whole wine consumption on health is still an open question, it is possible to speculate that a new field of research is opening up for dealcoholised wine and that it will take decades to reach reasonable and shared conclusions.

The first issue concerns the debated role of alcohol. Its negative health effects were demonstrated in numerous in vitro and animal studies mainly considering the administration of alcohol in different concentrations. In other cases, epidemiological research studied mortality or the onset of various diseases associated with alcohol intake, including wine, arriving at contrasting conclusions. However, the role of alcohol as a component of wine has been less considered. The results of the study by Sato et al. [253] on male rats documented that polyphenolic compounds in red wine provide cardioprotective benefits through their ability to function as antioxidants, while the alcohol component confers cardioprotective effects by adapting the heart to oxidative stress. Boban et al. [254] highlighted that antimicrobial activity of intact wine was higher with respect to phenol-stripped wine and ethanol alone, concluding that the antimicrobial activity of complex solutions, such as intact wine, cannot be attributed to single constituents. More recently, Liberale et al. [255] reviewed the updated knowledge on the risk of adverse cardiovascular events underlying the cardioprotective effects of polyphenols and ethanol. This concept has been taken up several times over the years, with conflicting results between authors who have claimed that there is a balance between alcohol and wine polyphenols that, in concert, would be responsible for the cardioprotective benefits and those who have argued that even low/moderate wine consumption could have a detrimental effect on health [256,257,258]. Notably, a consensus has not been reached, although there is broad epidemiological support for low to moderate drinking patterns.

#### 5.4.2. Low- and No-Alcohol Wine Stability

Ethanol is essential for the stability, ageing, and sensory properties of wine [52]. Its inhibitory activity, combined with the acidity of the wine and the addition of sulphur dioxide, allows the wine to remain sound for years in the absence of air. Ethanol also acts as a reagent in the formation of volatile compounds produced during fermentation and barrel ageing. Together with other alcohols, it reacts slowly with organic acids to produce esters and influences their stability. Given these premises, the microbial stability of low-alcohol and alcohol-free wines will be quite different in comparison with whole wines. In particular, in bottled dealcoholised wine, the lower alcohol content and any added sugars to improve its sensory attributes may facilitate refermentation processes [259], thus requiring the addition of further amounts of preservatives, such as sulphur dioxide and sulfiting agents. The maximum content of these substances in wine is regulated by specific legislation in different countries because of the risks to human health caused by their consumption [260,261]. In light of these limits, other preservative or stabilising agents could be adopted in low- or dealcoholised wine, such as some weak acids or their salts (sorbic and benzoic acid) and dimethyl dicarbonate [259]. Other strategies may include the use of non-*Saccharomyces* yeast strains able to produce antimicrobial compounds [262]. However, the mechanisms of action of these substances are relatively known in wine, while their possible synergistic effect with other added preservatives or compounds naturally present in these beverages must be evaluated. In particular, their effects should be carefully assessed in relation to human health.

#### 5.4.3. What Is Admitted in the Production of Low- and No-Alcohol Wine?

As reported before, dealcoholisation processes can variably influence the phenolic composition of wine, with a negative impact on its sensory quality and potential health benefits. In particular, the development of certain technologies to reduce alcohol content can lead, in some case, to the production of high-quality dealcoholised wines [238], but, in other cases, it may not. This mostly depends on the type of wine, the percent of ethanol removed, the type of dealcoholisation technique, and the operating conditions, which dramatically impact the total polyphenol content, the volatile profile, and the composition of the original wines [7]. Given this connection, it is quite obvious that global health policies will have to protect the consumer with clear rules on the quality of wines that can undergo dealcoholisation, the admission of additives to improve the taste and health impact of dealcoholised wine, and clear labelling to inform the consumer about what they are drinking and the origin of possible added ingredients.

## 6. Conclusions

In this review, we examined the scientific literature concerning the pros and cons of wine consumption in relation to human health. Dividing our search into two different periods, we found topics predominantly covered in the period from 1970 to 2000 and after 2000 to the present. Much of the research until 1990 comprised epidemiological studies focusing on alcohol abuse from various alcoholic beverages (wine, beer, spirits) and the health effect in relation to age, gender, and socio-economic conditions. However, several authors also reported observations on the inverse relationship between coronary heart disease and regular consumption of alcoholic beverages. Around the late 1980s to the early 1990s, it was clear that many of wine’s polyphenols had positive effects not only on wine’s quality but also on health. Wine, particularly red wine, has gradually become the protagonist in scientific research, and phenolic compounds were recognised for their potent antioxidant and anti-inflammatory effects. Since 2000, only a few new phenols have been reported, which are mainly derived from previously documented ones. Furthermore, it was shown that the compounds responsible for health effects are not only those ingested but also their metabolites, which are formed after absorption or after the action of the intestinal microbiota. The importance of wine consumption during meals and as part of a balanced diet was also emphasised. The attention of scientists over the years has remained high in relation to the alcohol content of wine and its possible negative effects on health. The controversies regarding the U-shaped (J-shaped) curve between alcohol consumption and mortality have continued, and the debate among scientists is still open. In this scenario, the market has recently proposed a new product line consisting of wines with low and no alcohol content, whose production technology is still under consideration.

Through the analysis of the literature over half a century of research, we could not find clear scientific evidence on the harmful role of low/moderate wine consumption in health. On the other hand, we did not find a clear position on the health benefits of low/moderate wine consumption. The same consideration can be made for low-alcohol and dealcoholised wines, which we considered in the last part of our work and for which several health concerns have been raised.

In light of the above considerations, we believe that the only conclusion we can draw is that moderate consumption of alcoholic wine, as well low-alcohol and dealcoholised wines, can all provide benefits for human health. In our opinion, information campaigns on the risks of alcohol consumption mainly refer to its abuse, and this is a correct and sustainable approach. However, the “alcohol zero” policy proposed by part of the scientific community cannot be supported, as much scientific evidence does not confirm this view. At long last, a new chapter of research on low-alcohol and dealcoholised wines has opened, giving rise to new and exciting studies that will keep winemakers and scientists involved for decades to come.

## Data Availability

No new data were created or analyzed in this study. Data sharing is not applicable to this article.
